# Neurobasal medium enhances titan cell formation in *Cryptococcus* spp.

**DOI:** 10.1590/0074-02760240286

**Published:** 2025-06-27

**Authors:** Juliana Godoy, Igor Avellar-Moura, Juliana Soares, Bruno Pontes, Susana Frases

**Affiliations:** 1 Universidade Federal do Rio de Janeiro, Instituto de Biofísica Carlos Chagas Filho, Laboratório de Biofísica de Fungos, Rio de Janeiro, RJ, Brasil; 2 Universidade Federal do Rio de Janeiro, Instituto de Ciências Biomédicas & Centro Nacional de Biologia Estrutural e Bioimagem, Laboratório de Pinças Ópticas, Rio de Janeiro, RJ, Brasil; 3 Rede Micologia Rio de Janeiro, Fundação de Amparo à Pesquisa do Estado do Rio de Janeiro, Rio de Janeiro, RJ, Brasil

**Keywords:** C. neoformans, C. gattii, titan cells, Neurobasal medium, virulence

## Abstract

**BACKGROUND:**

Titan cells in *Cryptococcus* species play a critical role in fungal virulence by resisting oxidative stress, phagocytosis, and antifungal treatments. Developing reliable methods to induce titan cells is crucial for understanding the mechanisms of *Cryptococcus* pathogenesis.

**OBJECTIVES:**

In this study we report an unexpected discovery of a simple *in vitro* induction of titan cells in *Cryptococcus neoformans* and *Cryptococcus gattii* using Neurobasal™ (NB) medium.

**METHODS AND FINDINGS:**

By employing established *in vitro* culture methods, we demonstrate a significantly higher capacity for titan cell formation in *Cryptococcus* spp. Cells grown in complete NB medium exhibited larger cell bodies, increased capsule sizes, and a higher percentage of titan cells compared to those grown in minimal medium (MM). NB medium without the B27 supplement significantly impacted titan cell formation.

**MAIN CONCLUSIONS:**

Our findings indicate that NB medium, originally developed for neuronal cell cultures, is a useful tool for studying titan cell biology. This is particularly relevant given the association between titan cells and the central nervous system, highlighting their potential role in *Cryptococcus* pathogenesis.

*Cryptococcus* spp., including *Cryptococcus neoformans* and *Cryptococcus gattii*, are opportunistic fungal pathogens that cause infections primarily in immunocompromised individuals, such as those with human immunodeficiency virus/acquired immunodeficiency syndrome (HIV/AIDS) or transplant recipients.^1^ ^,^ ^2^ This fungal pathogen is recognised for causing cryptococcal meningoencephalitis, a severe and often fatal manifestation of cryptococcosis, which is an opportunistic fungal infection responsible for approximately 181,000 deaths annually worldwide, primarily in sub-Saharan Africa. In addition to pulmonary infections, cryptococcal meningoencephalitis presents a significant threat to affected individuals. The pathogenesis of *Cryptococcus* involves complex interactions with central nervous system (CNS) cells; however, these mechanisms remain poorly understood despite extensive research efforts.^1^ ^,^ ^2^ These fungi are particularly noted for their ability to produce a polysaccharide capsule, which serves as a key virulence factor by protecting the organism from host immune defences, facilitating immune evasion, and resisting environmental stress.^3^ ^,^ ^4^ ^,^ ^5^

In addition to their capsule, *Cryptococcus* spp. exhibit a range of morphological adaptations that contribute to their pathogenicity.^4^ ^,^ ^6^ One such adaptation is the formation of titan cells, which are large, polyploid cells with diameters exceeding 10 µm.^6^ ^,^ ^7^ ^,^ ^8^ Titan cells are thought to be involved in the fungus’s ability to survive under hostile conditions, such as oxidative stress, and to evade immune responses.^9^ ^,^ ^10^ ^,^ ^11^ ^,^ ^12^ These cells also contribute to the persistence of *Cryptococcus* spp. within the brain.^13^

Despite their relevance to pathogenesis, the study of titan cells has been hindered by the challenges in their *in vitro* induction. Different culture media and conditions have been tested to promote titan cell formation.^12^ ^,^ ^14^ ^,^ ^15^ ^,^ ^16^ While some protocols involve the use of minimal media (MM) to induce stress and capsule growth,^15^ others have incorporated mammalian cell culture media, serum, phospholipids, and low-oxygen conditions to stimulate titan cell formation.^12^ ^,^ ^14^ ^,^ ^16^ The development of optimised culture conditions for titan cells is essential for understanding their role in *Cryptococcus* biology and virulence.

In this study, we present a simple *in vitro* approach using Neurobasal™ (NB) medium as an alternative to induce titan cell formation in *C. neoformans* and *C. gattii*. We analyse the effects of NB medium on cell size, capsule formation, and the percentage of titan cells produced, providing a reliable framework for studying these cells in the laboratory and opening possibilities for exploring their role in *Cryptococcus* pathogenesis.

## MATERIALS AND METHODS

*Strains and culture conditions* - The *C. neoformans* var. grubii H99 (ATCC 20882) was used as a standard strain for this study. Additionally, *C. gattii* L25/01 was provided by Prof Daniel de Asis Santos (Federal University of Minas Gerais, Brazil). The yeasts were cultured in both MM and NB medium. MM consisted of 15 mM glucose, 10 mM MgSO₄, 29 mM KH₂PO₄, 13 mM glycine in ultrapure MilliQ water, pH 5.5 ± 0.1, sterilised by autoclaving. MM was used as a control to induce nutritional stress and capsule growth, following the protocol by Zaragoza et al.^17^ NB medium (Thermo Fisher Scientific, USA) was supplemented with 2 mM L-glutamine, 1% penicillin/streptomycin, and 1% B-27 supplement (Gibco Scientific, USA - catalog number #17504001), used to support neuronal cell differentiation.^18^ Some experiments were also conducted in NB medium without the B27 supplement, but with all other reagents included. The B27 supplement is a complex mixture comprising several essential components: various vitamins, including A, E, D3, K, and B-complex vitamins (B1, B2, B6, B12); important proteins and hormones such as insulin, transferrin, and progesterone; other crucial molecules, including selenium, biotin, L-carnitine, and ethanolamine; antioxidants like glutathione and superoxide dismutase; and fatty acids, including linoleic acid, linolenic acid, and corticosterone. For more information on all compounds in the B27 supplement used, please refer to: https://www.thermofisher.com/br/en/home/technical-resources/media-formulation.250.html.

*Pre-inoculum preparation* - A 10 mL pre-inoculum of each fungal strain was prepared by inoculating *C. neoformans* and *C. gattii* into liquid Sabouraud medium and incubating at 37ºC with constant agitation (150 rpm) for 24 h. The culture was then harvested by centrifugation (6,708 x g, 10 min), and the cells were resuspended in phosphate buffered saline (PBS) for further experiments.

*Inoculation and incubation* - The fungal cell suspension was quantified using a Neubauer chamber. A 6-well plate was prepared with 2 mL of each tested medium (NB and MM) per well, inoculated with 5 × 10³ fungal cells per mL. The plates were incubated at 37ºC and 5% CO_2_ for five days. CO₂ was used to maintain the proper pH of NB medium, as pH regulation is essential for its function.

*Morphological analysis* - To assess cell morphological parameters, including capsule size, cell body diameter, and total cell size, a negative staining method using India ink was employed, as described by Zaragoza et al.^17^ The process involved centrifuging 1 mL of yeast cell suspension from each medium at 5,000 rpm for 10 min, followed by resuspension in 1 mL of PBS. Subsequently, slide mounting was performed using 5 μL of *Cryptococcus* spp. cells resuspended in PBS, mixed with 5 μL of India ink. Visualisation of the prepared slides was conducted using the Axio Observer microscope (Zeiss, Jena, Germany). Our criteria for categorising cells as titans were based on a cell size threshold of 10 µm or larger. For further characterisation of cell morphometry, random images containing a minimum of 100 cells were captured and analysed using ImageJ 1.8.0g software (http://rsb.info.nih.gov/ij/) provided by the National Institutes of Health (NIH, Bethesda, MD, USA).

*Statistical analysis* - All data obtained were subjected to statistical analysis by Student’s t-test using the GraphPad Prism version 10.0.1 (San Diego, California, USA). Results were considered statistically significant with a p-value ≤ 0.05.

## RESULTS

While conducting *in vitro* co-culture experiments of *C. neoformans* with differentiated neuronal cell cultures, we unexpectedly observed that fungal cells appeared significantly larger than usual. This surprising finding led us to better explore whether NB medium could act as an inducer of titan cells in *Cryptococcus* spp.

**Fig. 1:**
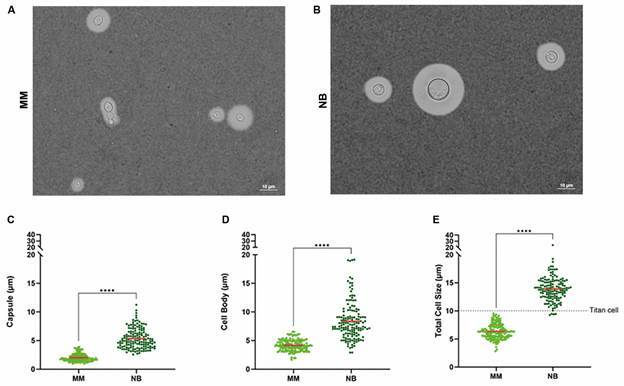
Neurobasal (NB) medium induces formation of titan cells in *Cryptococcus neoformans*. (A-B) Representative images of *C. neoformans* cells stained with India ink under optical microscopy in minimal media (MM) (A) and NB medium (B), Scale bar: 10 μm. (C-E) Scatter plots showing the quantification of capsule (C), cell body (D), and total cell size (E) of *C. neoformans* cells cultured in MM (light green) or NB medium (dark green). The experiments were performed on three different occasions, and at least 150 cells from different fields were used for quantification. ****indicate p-value < 0.0001 using Student’s t-test statistics.

Thus, *C. neoformans* was cultured in complete NB medium for five days at 37ºC and 5% CO_2_ atmosphere. The 5-day timepoint was selected because, at this stage, the culture reaches the stationary phase, providing a homogeneous population suitable for the overall analysis. Morphological parameters were assessed by measuring capsule size, cell body diameter, and total cell size using India ink staining (Fig. 1A-B). The results showed significant increases in all parameters when yeast cells were grown in complete NB medium compared to MM (Fig. 1). Specifically, capsule size increased by 2.7-fold (Fig. 1C), cell body diameter by 2.0-fold (Fig. 1D), and the total cell size by 2.2-fold (Fig. 1D) in complete NB medium relative to MM.

**Fig. 2:**
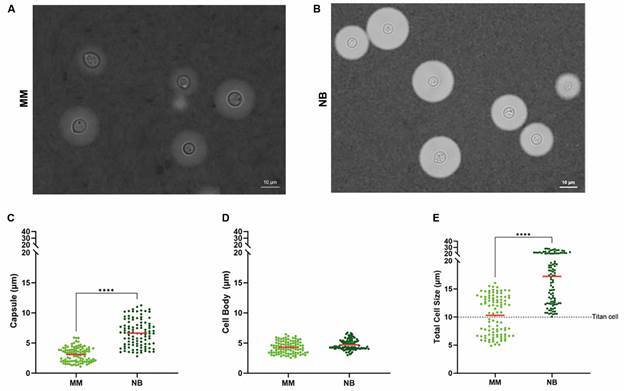
Neurobasal (NB) medium induces formation of titan cells in *Cryptococcus gatti*. (A-B) Representative images of *C. gatti* cells stained with India ink under optical microscopy in minimal media (MM) (A) and NB medium (B), Scale bar: 10 μm. (C-E) Scatter plots showing the quantification of capsule (C), cell body (D), and total cell size (E) of *C. gatti* cells cultured in MM (light green) or NB medium (dark green). The experiments were performed on three different occasions, and at least 100 cells from different fields were used for quantification. ****indicate p-value < 0.0001 using Student’s t-test statistics.

Similar experiments were also conducted with *C. gattii* (Fig. 2). The results showed significant increases in capsule size, 2.1-fold (Fig. 2C), and total cell size, 1.7-fold (Fig. 2E), although the cell body diameter did not exhibit marked differences in complete NB medium when compared to MM (Fig. 2C).

**Fig. 3:**
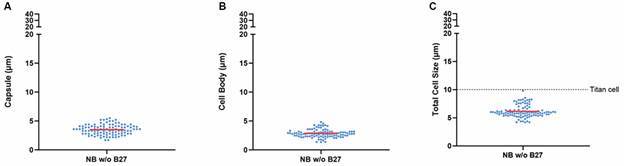
removal of B27 supplement from Neurobasal (NB) medium impairs formation of titan cells in *Cryptococcus neoformans*. Scatter plots showing the quantification of capsule (A), cell body (B), and total cell size (C) of *C. neoformans* cells cultured in NB medium without B27 supplement (light blue). The experiments were performed on three different occasions, and at least 100 cells from different fields were used for quantification.

NB medium is supplemented with 1% B27, a mixture of different compounds known to promote growth, differentiation, and maintenance of neuronal cells *in* vitro.^19^ To investigate the role of B27 in the observed effects on *Cryptococcus* cells, we next removed B27 from the NB medium. The results (Fig. 3) revealed a marked reduction in all measured morphological parameters, including capsule size (Fig. 3A), cell body diameter (Fig. 3B), and total cell size (Fig. 3C), when compared to cultures grown in complete NB medium (with B27) (Fig. 2), suggesting that B27 supplementation plays a crucial role in titan cell induction in *Cryptococcus* spp.

Finally, to quantify titan cell formation, a size threshold of 10 µm for total cell size was applied. In complete NB medium, 97% of *C. neoformans* cells (Fig. 1E) and 100% of *C. gattii* cells (Fig. 2E) were classified as titan cells. In contrast, 0% and 53% of cells respectively from both species grown in MM met the titan cell criteria (Figs 1E, 2E). When NB medium was used without B27 supplementation, 0% of cells were classified as titan cells in *C. neoformans* (Fig. 3C). These results demonstrate that complete NB medium is an effective inducer of titan cells in both *C. neoformans* and *C. gattii*, providing a valuable tool for studying the role of titan cells in *Cryptococcus* pathogenesis.

## DISCUSSION

The formation of titan cells in *Cryptococcus* spp. has been recognised as a key factor in the pathogenicity of these fungi. Titan cells are characterised by their large size and resistance to host immune responses, including phagocytosis, oxidative stress, and antifungal treatments.^9^ ^,^ ^10^ ^,^ ^11^ ^,^ ^12^ ^,^ ^20^ These traits are thought to contribute significantly to the ability of *Cryptococcus* to cause severe infections, especially in immunocompromised individuals. Despite their importance, the induction of titan cells *in vitro* has posed challenges, and reliable culture conditions for their study remain limited.^12^ ^,^ ^14^ ^,^ ^15^ ^,^ ^16^

In the present study, we serendipitously discovered that NB medium, originally designed for culturing neuronal cells, effectively promotes the formation of titan cells in *C. neoformans* and *C. gattii*. Yeast cells cultured in complete NB medium showed significant increases in cell body, capsule, and total cell sizes compared to those grown in MM. These morphological changes observed in our study align with the characteristics of titan cells, which are typically larger than regular yeast cells.^6^ ^,^ ^7^ ^,^ ^8^ We hypothesise that the nutrient-rich composition of complete NB medium and its ability to replicate the conditions of the CNS - a common site of *Cryptococcus* colonisation - are key factors driving this differentiation.^21^

Brain infection by *C. neoformans* is highly fatal. Cryptococcal meningoencephalitis, a fatal form of cryptococcosis, is responsible for 181.000 deaths per year globally, mostly in sub-Saharan Africa.^22^ Despite extensive research, the interactions between *Cryptococcus* and CNS cells, and the role of factors like glucuronoxylomannan (GXM) in pathogenesis, remain poorly understood. Additionally, the lack of effective *in vitro* models to simulate brain-fungal interactions hinders progress in the field.^23^ ^,^ ^24^ Titan cells have been observed in CNS *in vivo*,^13^ ^,^ ^21^ ^,^ ^25^ and complete NB medium is designed to mimic the CNS microenvironment for *in vitro* neuronal growth.^19^ ^,^ ^26^ These correlations strongly support that the conditions offered by complete NB medium closely resemble those in the brain, thereby promoting the differentiation of titan cells in *C. neoformans* and *C. gattii* and allowing more detailed *in vitro* studies of their role in neurocryptococosis. In addition, the use of NB medium to generate these cells offers a valuable model for studying their molecular and cellular properties, as well as their physicochemical and biological parameters, including capsule viscosity, elasticity, composition, and fungal resistance to oxidative stress, to antifungal drugs, and to immune response.

Moreover, complete NB medium is rich in nutrients, including glutamine, vitamins, and neurotrophic factors, which are known to support neuronal cell growth and differentiation.^18^ ^,^ ^19^ ^,^ ^26^ Studies have shown that growth factors, such as neurotrophic factors and vitamins, can modulate the growth of pathogenic fungi.^7^ Thus, indicating that NB medium might mimic the signalling pathways involved in titan cell differentiation. Notably, the removal of B27 from the NB medium reduced the percentage of titan cells in our study, suggesting that B27 is essential for optimal titanisation. We hypothesise that the mixture of compounds in B27 works synergistically, rather than through a single factor, to promote titan cell formation, as it does for neuronal cell survival and growth *in vitro*. Our observations were all performed after the 5-day timepoint. At this stage, the culture reaches stationary phase, with cells experiencing nutrient depletion, stress, and stabilisation, resulting in a homogeneous population.^5^ Although titan cells could theoretically form earlier, the 5-day timepoint allows sufficient time for the accumulation of quorum sensing molecules and other factors that might influence the titanisation process.^14^ ^,^ ^15^

In conclusion, our findings demonstrate that NB medium is an effective tool for *in vitro* induction of titan cells in *C. neoformans* and *C. gattii*. The significant increase in titan cell formation observed in NB medium underscores its potential for studying the role of titan cells in *Cryptococcus* pathogenesis. Finally, our results contribute to the expanding knowledge on titan cell biology and provide a promising approach for future research aimed at more systematically investigating the mechanisms that drive virulence in these human pathogens.
